# 5,6-Dimethylxanthenone-4-acetic acid (DMXAA), a novel antivascular agent: phase I clinical and pharmacokinetic study

**DOI:** 10.1038/sj.bjc.6600885

**Published:** 2003-04-15

**Authors:** G J S Rustin, C Bradley, S Galbraith, M Stratford, P Loadman, S Waller, K Bellenger, L Gumbrell, L Folkes, G Halbert

**Affiliations:** 1 Department of Medical Oncology, Mount Vernon Hospital, Northwood, Middlesex HA6 2RN, UK; 2 Bradford Royal Infirmary, Bradford BD9 6RJ, UK; 3 Gray Cancer Institute Mount Vernon Hospital, Northwood, Middlesex HA6 2JR, UK; 4 Cancer Research Unit, University of Bradford, Bradford BD7 1DP, UK; 5 Drug Development Office of Cancer Research UK, PO Box 123, London WC2A 3PX, UK; 6 Cancer Research UK Formulation Unit, University of Strathclyde, Glasgow G1 1XW, UK

**Keywords:** DMXAA, pharmacokinetics, pharmacodynamics, toxicity

## Abstract

The purpose of this phase I, dose-escalation study was to determine the toxicity, maximum tolerated dose, pharmacokinetics, and pharmacodynamic end points of 5,6-dimethylxanthenone acetic acid (DMXAA). In all, 46 patients received a total of 247 infusions of DMXAA over 15 dose levels ranging from 6 to 4900 mg m^−2^. The maximum tolerated dose was established at 3700 mg m^−2^; dose-limiting toxicities in the form of urinary incontinence, visual disturbance, and anxiety were observed at the highest dose level (4900 mg m^−2^). The pharmacokinetics of DMXAA were dose dependent. Peak concentrations and area under the curve level increased from 4.8 *μ*M and 3.2 *μ*M h, respectively, at 6 mg m^−2^ to 1290 *μ*M and 7600 *μ*M h at 3700 mg m^−2^, while clearance declined from 7.4 to 1.7 l h^−1^ m^−2^ over the same dose range. The terminal half-life was 8.1±4.3 h. More than 99% of the drug was protein bound at doses up to 320 mg m^−2^; at higher doses the percent free drug increased to a maximum of 6.9% at 4900 mg m^−2^. Dose-dependent increases in the serotonin metabolite 5-hydroxyindoleacetic acid were observed at dose levels of 650 mg m^−2^ and above. There was one unconfirmed partial response at 1300 mg m^−2^. In conclusion, DMXAA is a novel vascular targeting agent and is well tolerated.

While research interest in cancer therapy that targets tumour vasculature is currently directed at angiogenesis inhibitors, agents that interrupt existing tumour vasculature are also under investigation. The antitumour activity of flavone acetic acid (FAA) against a range of transplantable murine tumours with established vasculature is well documented (Plowman *et al*, 1986; Smith *et al*, 1987; Hill *et al*, 1989) and appears to be related to the production of tumour necrosis factor-alpha (TNF-*α*), since antibodies to TNF can prevent the reduction in blood flow caused by FAA (Mahadevan *et al*, 1990). In contrast to its activity in murine cell lines, FAA does not induce TNF in human mononuclear cell lines (Ching *et al*, 1994), and species differences in the target of action are the most likely explanation for the apparent failure of FAA to demonstrate any significant clinical antitumour activity.

A research programme at the Auckland Cancer Society Research Centre (Auckland, New Zealand) directed at synthesising analogues of FAA without a species difference in antitumour activity resulted in 5,6-dimethylxanthenone acetic acid (DMXAA) (Rewcastle *et al*, 1989, 1991). More active than FAA, and with a 12-fold higher potency in murine tumour models (Rewcastle *et al*, 1991), DMXAA induces TNF production in cell lines from humans, as well as those from mice. The mechanism of antitumour action of DMXAA, like that of FAA, differs from that of directly cytotoxic drugs. 5,6-Dimethylxanthenone acetic acid has selective activity in targeting tumour vasculature *in vivo*, producing vascular shutdown, reducing tumour blood flow, and consequently causing haemorrhagic necrosis (Zwi *et al*, 1994b; Laws *et al*, 1995).

Repeat dosing at 3- or 7-day intervals produced greater induction of TNF-*α* and growth delay in colon 38 tumours in mice than a single dose of DMXAA (Philpott *et al*, 1995). A single- and multiple-dose toxicity study was therefore performed on MF1 mice and Wistar rats, which indicated that the dose that was lethal in 10% (LD_10_) was 20 mg kg^−1^ (BIBRA International, Carshalton, Surrey, UK). The starting dose in humans is based on 10% of this figure equivalent to 6 mg m^−2^. The Cancer Research Campaign (now Cancer Research UK) has conducted two parallel phase I trials of DMXAA. A single centre study in Auckland, New Zealand used a 3-weekly schedule (Jameson *et al*, submitted). A two-centre UK study, at Mount Vernon Hospital, Northwood and Bradford Royal Infirmary, Bradford, used a weekly schedule and is the subject of this report. The primary objectives of this study were to: (1) determine the toxicity profile of DMXAA, (2) establish the maximum tolerated dose (MTD), (3) characterise the pharmacokinetic profile, and (4) determine the effects of DMXAA on coagulation factors and the production of TNF-*α*, nitric oxide, and serotonin. A secondary aim was to assess the antitumour efficacy of DMXAA. Dynamic contrast enhanced magnetic resonance imaging (DCE-MRI) studies to assess the effect of DMXAA on human tumour vasculature were incorporated into the two phase I trials by a protocol amendment and the results of these studies are reported in full separately (Galbraith *et al*, 2002).

## PATIENTS AND METHODS

### Eligibility

Male or female patients aged 18–75 years with histologically confirmed cancer not amenable to standard curative therapy or refractory to conventional therapy were eligible for inclusion in this study. Other eligibility criteria included documented disease progression within 2 months prior to entry into the study, a World Health Organisation (WHO) performance status of 0–2, a life expectancy of >3 months, and adequate bone marrow, hepatic, and renal function. Patients who had previously received anticancer therapy were eligible if at least 4 weeks had elapsed since completion of that therapy (6 weeks for nitrosoureas and mitomycin C). Patients were excluded from the study if they had concurrent malignancies, or if they had experienced any other serious medical conditions, uncontrolled or serious infection in the 28 days prior to the study. Women who were capable of child bearing were required to use effective contraceptive methods. The local ethics committees approved the study, and all patients gave written informed consent prior to participation in the study.

### Study design

5,6-Dimethylxanthenone acetic acid was formulated as a 20-mg ml^−1^ solution in 0.1 M phosphate buffer at pH 7.7 in amber glass vials and stored at room temperature. During the study, the formulation was changed to 100 mg ml^−1^ in 0.02 M phosphate buffer at pH 7.9 to accommodate higher doses while minimising infusion volume. Patients treated at 3700 and 4900 mg m^−2^ received the 100 mg ml^−1^ formulation.

5,6-Dimethylxanthenone acetic acid was administered as a 20-min intravenous infusion via a peripheral vein in a once-weekly schedule. The starting dose of DMXAA was 6 mg m^−2^ and dose escalation was planned to follow a modified Fibonacci scheme subject to toxicity and pharmacokinetic data. It was planned that if DMXAA area under the curve (AUC) levels were lower than expected from the animal data, then dose escalation would be accelerated. Three patients were entered at each dose level and two patients had to be followed for at least 3 weeks before any patients could be entered at the next level. If any of the following toxicities occurred, three additional patients were to be entered: >Grade 2 neutropenia, mucositis, diarrhoea, alanine aminotransferase serum (ALT), aspartate aminotransferase serum (AST), renal toxicity, vomiting that did not respond to symptomatic treatment, >Grade 1 thrombocytopenia, neurological or cardiac toxicity, or an increase of one grade of peripheral neuropathy, if present at baseline. Where specific toxicities were not considered to be drug related, dose escalation was continued without expanding the dose level.

Before treatment and once weekly during the study, patients were given a physical assessment (including WHO performance status), and routine laboratory tests including full blood count (FBC) with differential, international normalised ratio (INR), activated partial thromboplastin time (APTT), and urine analysis were performed. Full blood count was repeated 24 and 72 h after the first infusion and 24 h after the fourth infusion. An electrocardiogram (ECG) was performed before treatment and at the end of the study. During treatment, blood pressure and pulse were taken at 5 and 15 min during the infusion, at the end of the infusion, and at 30-min intervals from the end of the infusion, for at least 6 h.

The National Cancer Institute of Canada Clinical Trials Group (NCIC-CTG) Expanded Common Toxicity Criteria (CTC) were used to assess adverse events and their severity. Dose-limiting toxicity (DLT) was defined as Grade 2 neurotoxicity, Grade 3 for other nonhaematological toxicity (excluding alopecia and nausea), or Grade 4 haematological toxicity, which lasted more than 4 days or was associated with fever or bleeding. The MTD was defined as the dose below that at which more than 30% of the patient population experienced unacceptable toxicity caused by DMXAA.

One course of treatment consisted of six once-weekly infusions of DMXAA. If tumour response was observed or patients were benefiting from treatment, they could receive a maximum of two courses, otherwise they came off study after one course.

Disease measurements (CT scan or chest X-ray) were made at baseline and week 6 to assess response and again after 12 weeks if the patient remained on study. Response criteria were those produced by the WHO.

### Pharmacokinetic studies

Blood samples (7 ml) were collected into heparinised tubes immediately before the start of the infusion, immediately before the end of the infusion (*t*=0), and at 0.25, 0.5, 0.75, 1, 1.5, 2, 4, 8, 12, and 24 h after the end of the infusion for the first dose of DMXAA. Following subsequent infusions in the same patient, samples were taken before treatment, immediately before the end of the infusion, and at 4 h after the end of treatment. The blood was centrifuged at 1500 **g** for 10 min and aliquots of plasma were stored at −20°C. Plasma concentrations of DMXAA were determined using high-performance liquid chromatography (HPLC) with fluorescence detection. As a result of the almost three orders of magnitude dose range, exact amounts of sample and internal standard (i.s.) varied according to the expected concentration range, but typically to 100 *μ*l plasma was added 50 *μ*l i.s. (5-propoxyxanthenone acetic acid, 100 nmol ml^−1^), the sample was mixed, and 300 *μ*l acetonitrile : methanol (3 : 1 v v^−1^) added. After again mixing, the protein precipitate was removed by centrifugation (12 000 **g**, 1 min), and the supernatant dried in a centrifugal evaporator (Hetovac). The samples were reconstituted in 120 *μ*l HPLC starting eluent and transferred to HPLC vials for injection. For samples from doses above 1000 mg m^−2^, the drying stage was omitted. The assay was validated using control human plasma spiked with known amounts of DMXAA, and found to be linear from 50 nM to 2 mM. Intra- and interassay recovery was between 102 and 104% and CV ranged from 0.2% at 20 *μ*M to 1.9% at 500 *μ*M.

Free plasma DMXAA concentrations were determined by ultrafiltration. Samples of plasma (300 *μ*l) were added to Amicon Centrifree YM-30 filters, centrifuged at room temperature (2000 **g**, 20 min), and the resultant filtrate analysed without further processing by HPLC.

Separation of DMXAA from its metabolites and the i.s. was achieved using a linear gradient. The column was a Lichrospher RP18e cartridge (125 × 4 mm, Merck, Aston Scientific Stoke Mandeville, UK), fitted with a guard column (4 × 4 mm) packed with the same material. Eluents comprised (A) 2% (v v^−1^) acetic acid (HiPerSolv, Merck, Aston Scientific Stoke Mandeville, UK) and (B) acetonitrile : water (3 : 1 v v^−1^), and the gradient was run from 35 to 62% B over 9 min, then increased to 80% over 0.5 min, and held there for 3 min before returning to the starting conditions. For free drug determinations, only the first stage of the gradient was required. The flow rate was 1.6 ml min^−1^. Detection was by fluorescence using a Waters 474 detector fitted with a 5 *μ*l flow cell, with excitation at 345 nm, emission 409 nm. Samples were injected using a Waters 717 autosampler, and data were collected and processed using Waters Millennium software. Guard columns were changed every ∼200 analyses.

Pharmacokinetic data were fitted using nonlinear least-squares analysis, with statistical weighting inversely proportional to concentration^2^, to a mono-, bi-, or tri-exponential elimination function using a computer-based fitting program (Microcal Origin, Aston Scientific Stoke Mandeville, UK). The most appropriate function was chosen by minimising Akaike's information criterion (AIC) (Yamaoka *et al*, 1978). The AUC was calculated using the trapezoid method to give the value to 24 h, then extrapolating to infinity by adding *C*_24h_/*k*_el_. Clearance was calculated as dose/AUC.

### Pharmacodynamic studies

Blood samples for the determination of nitrate and TNF-*α* were collected as outlined in the pharmacokinetics section and were stored at −20 and −70°C for nitrate and TNF-*α*, respectively. Additional samples for 5-hydroxyindoleacetic acid (5-HIAA) determination were taken pretreatment and at 1, 4, 8, and 24 h postinfusion. Samples were stored at −70°C. Plasma concentrations of nitrate were determined by ion-exchange chromatography with absorbance detection (Stratford, 1999). Nitrate is the end product of nitric oxide synthesis, and is raised in mice after treatment with DMXAA (Thomsen *et al*, 1991; Moilanen *et al*, 1998). Plasma TNF-*α* concentrations were determined using a commercially available ELISA kit (Genzyme). Serotonin mediates the effect of TNF-*α* and has been shown to be important in the action of DMXAA (Baguley *et al*, 1993). Its metabolite 5-HIAA was measured as serotonin is too unstable. Concentrations of 5-HIAA in plasma were determined by HPLC with electrochemical detection (Kestell *et al*, 2001).

By a protocol amendment, DCE-MRI scans were performed on patients treated with DMXAA at doses of 500 mg m^−2^ or higher and are reported separately (Galbraith *et al*, 2002).

## RESULTS

### Patient characteristics

In total, 46 patients were recruited to the study. All but one had undergone one or more surgical procedures. Their baseline characteristics and prior therapy are summarised in Table 1

**Table 1:** Patient characteristics and prior therapy

**Characteristic**	**Patients (*n*)**
No. of patients (M/F)	46 (23/23)
Mean age (range) in years	58 (34–77)
*WHO performance status*	
0	13
1	29
2	4
	
*Tumour types*	
Colon/rectum	9
Kidney	8
Ovary	7
Unknown primary	7
Melanoma	4
Soft tissue sarcoma	3
Carcinoid/small bowel	2
Uterus	2
Breast	1
Lung	1
Oesophagus	1
Pancreas	1
Renal pelvis	1
	
*Prior therapy*	
Radiotherapy	12
Biotherapy/hormone	11
Chemotherapy	35

.

All patients were included in the analysis of the toxicity of DMXAA and in the pharmacokinetic and pharmacodynamic evaluations; however, 10 patients were excluded from the assessment of response, including one patient who was excluded because she continued to take tamoxifen therapy. The 46 patients in the study received a total of 247 infusions of DMXAA, a median of six infusions per patient (range 1–11), given once weekly. One patient, treated at 650 mg m^−2^, was retreated as a new patient at 2300 mg m^−2^.

Table 2

**Table 2:** Dose-escalation schedule

**Dose level (mg m^−2^)**	**Patients per dose level (*n*)**	**Infusions (*n*)**
6	3	15
10.2	3	21
20.4	4^a^	18
40.8	4^a^	23
81.6	4^a^	24
160	3	18
320	3	17
500	3	12
650	3	17
1000	3	15
1300	3	16
1750	3	11
2300	3	18
3700	3	21
4900	1	1

a An amendment to the protocol required that four patients were treated at each dose level; however, the number of patients at each dose level returned to three some months later. One patient treated at 650 mg m^−2^ was retreated as a new patient at 2300 mg m^−2^.

presents the dose-escalation schedule, with the number of patients treated at each dose level, and the number of infusions of DMXAA they received.

The dose of DMXAA was escalated from 6 to 4900 mg m^−2^ in 15 dose levels. The first escalation in dose (1.7-fold increase) was carried out in line with the planned modified Fibonacci scheme. As plasma AUC values at the first dose level were lower than predicted for DMXAA, subsequent dose escalation was guided by a combination of toxicity and pharmacokinetic data, both from this study and the parallel phase I study in New Zealand (Jameson *et al*, submitted). There were five successive dose escalations that doubled the dose of DMXAA; further dose escalations increased the dose by factors of 1.3–1.6.

### Toxicity

After the fifth patient (treated at 10.2 mg m^−2^) became delirious following the fifth and sixth infusions of DMXAA, the protocol was amended to increase the number of patients at each dose level to four in order to obtain information about the cumulative toxicity of DMXAA. As no further cumulative toxicities were observed in the next 13 patients (10.2–81.6 mg m^−2^), the amendment was cancelled and the number of patients at each dose level returned to three.

The MTD was established at 3700 mg m^−2^ with three patients in the UK and seven patients in New Zealand receiving this dose. One patient with ovarian cancer was treated at the highest dose level (4900 mg m^−2^) when DLT was observed; the patient experienced Grade 3 urinary incontinence, Grade 2 visual disturbance, and Grade 2 anxiety. It was considered unethical to treat any more patients at 4900 mg m^−2^ as all three patients treated in New Zealand at that dose experienced DLT (MB Jamieson, personal communication). This consisted of one or more of tremor, feeling light-head and restless, urinary incontinence, Grade 2 visual disturbance anxiety, slurred speech, confusion and expressive dysphasia, and in one case breathlessness and left ventricular failure. Table 3

**Table 3:** Doses at which the most frequently reported drug-related toxicities were seen with DMXAA and their maximum grade per patient

	**Maximum toxicity grade**							
	**6–500 mg m^−2^ (*n*=27)**		**650–1300 mg m^−2^ (*n*=9)**		**1750 mg m^−2^ (*n*=3)**		**2300–4900 mg m^−2^ (*n*=7)**	
**Adverse event**	**Grade 1**	**Grades 2–4**	**Grade 1**	**Grades 2–4**	**Grade 1**	**Grades 2–4**	**Grade 1**	**Grades 2–4**
**Haematological**								
Haemoglobin	2	1	2		2		3	
Lymphocytes	1	2	1	3				4
PT		1		1			3	1
**Nonhaematological**								
*Neurological*								
Dizziness/vertigo	5				2		5	2
Headache	3	1	1		1			
Pain	1	3		3		1	4	
Other (inc. visual disturbance)	2		2		3		5	2
*Gastrointestinal*								
Nausea	12	2	1	1	2	1	2	
Vomiting	6	1		1		1	1	2
Anorexia	2	2	1				1	
*Cardiac*								
Hypertension	3		1		1		4	
Hypotension	4		1				1	
*Flu-like symptoms*								
Malaise	5	3	1	1			1	1
*Dermatological*								
Local		1			3		4	

PT=prothrombin time.

presents the most frequently reported toxicities that were considered possibly, probably, or almost certainly related to treatment with DMXAA.

There was no significant haematological toxicity such as neutropenia related to DMXAA. As expected in patients with advanced cancer, anaemia (Grades 1 and 2) and lymphocytopenia (mostly Grade 3) occurred commonly but were judged mostly to be unrelated to treatment.

The most common toxicities related to treatment were neurological symptoms, primarily visual disturbance, dizziness/vertigo, headache, and tumour pain. At dose levels up to and including 650 mg m^−2^, some visual disturbance was reported. One patient with colorectal cancer at a dose level of 6 mg m^−2^ experienced subtotal loss of vision (Grade 1) and other neurological symptoms (Grades 1–3). One patient with uterine cancer treated at 10.2 mg m^−2^ had spots before the left eye (Grade 1). Grade 1 disturbance of colour vision (±blurred or brightness of vision) was recorded at doses above 650 mg m^−2^. All patients who received doses of 1750 mg m^−2^ or higher experienced Grade 1–2 disturbance of colour vision following each infusion. The median time to onset was 10 min and the median duration 28.5 min with complete recovery. Grade 2 disturbance of colour vision was reported in one patient at 3700 mg m^−2^ and one patient at 4900 mg m^−2^.

Treatment-related tumour pain (Grades 1–3) was reported in 12 patients (26%) at doses of 40.8 mg m^−2^ and above. At 3700 mg m^−2^, one patient who had a total of 11 infusions of DMXAA experienced warmth/tightness in the tumour following the first, second, and eighth infusion, starting at 17–44 min after the infusion and lasting for 100–115 min. In total, 10 patients (22%) experienced Grade 1 dizziness/vertigo (including light-headedness) related to treatment. These symptoms were reported from the first dose level (6 mg m^−2^), but were of brief duration (median 15 min) and were not dose limiting.

Nausea (mainly Grade 1) and vomiting (Grades1 or 2) were the most frequently reported treatment-related gastrointestinal symptoms, affecting 21 (46%) and 12 (26%) of the patients, respectively. These symptoms were reported from the first dose level (6 mg m^−2^) but were of brief duration and not dose limiting. At higher dose levels, the time to onset was more acute, with nausea or vomiting occurring within minutes at a dose level of 1750 mg m^−2^ or higher. Patients who experienced nausea or vomiting following the first infusion received antiemetics prior to further infusions, which controlled vomiting but not nausea.

There were no major cardiovascular effects associated with DMXAA treatment. Nine patients (20%) experienced Grade 1 drug-related hypertension and Grade 1 drug-related hypotension was recorded in six patients (13%). Neither hypertension nor hypotension appeared to be dose dependent.

Malaise was the flu-like symptom most frequently attributed to treatment, occurring in 12 patients (26%; seven with Grade 1 symptoms). Malaise occurred at all dose levels and was not dose dependent, cumulative, or dose limiting. Grade 1 or 2 sweating was attributed to treatment in four patients (9%), with two patients experiencing the symptom in conjunction with the infusion at doses of 3700 mg m^−2^ or above. Seven patients (15%) experienced either Grade 1 venous irritation or pain at the site of intravenous injection, related to the infusion of DMXAA.

### Pharmacokinetics

**Figure 1:**
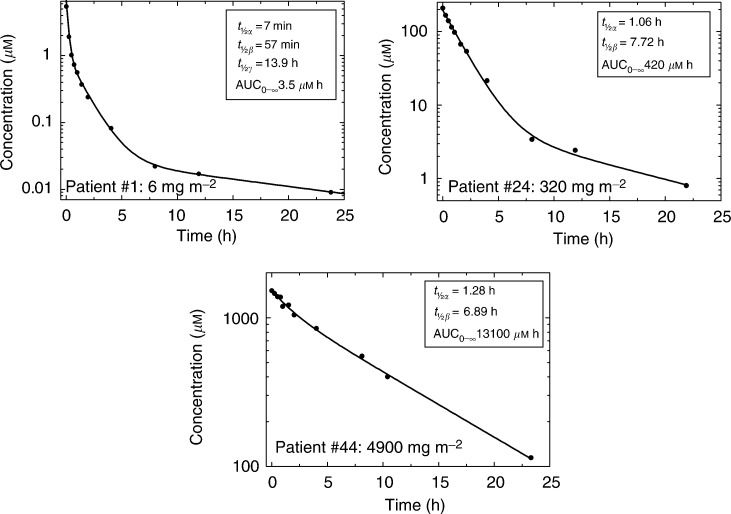
Plasma concentrations of DMXAA after 6, 320, and 4900 mg m^−2^.

There was a progressive change in the pharmacokinetic model with increase in dose of DMXAA (from a three- to a one-compartment model, Figure 1

). The terminal elimination half-life for all patients was 8.1±4.3 h.

**Figure 2:**
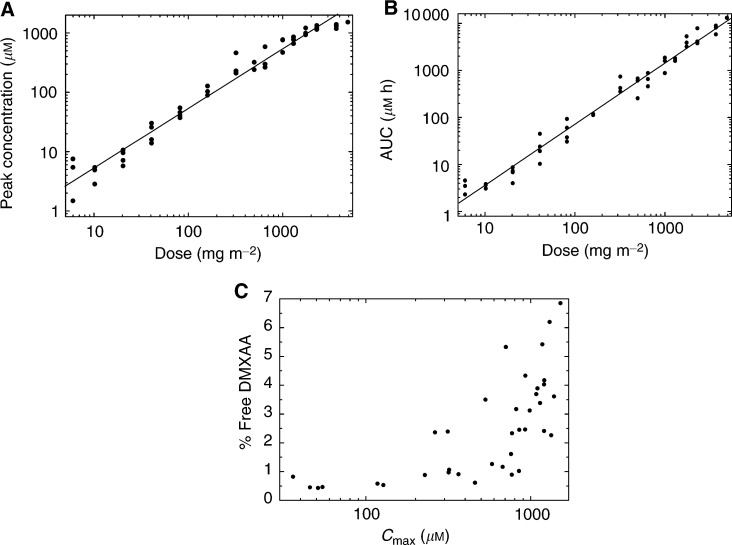
Relation between dose and (**A**) peak concentrations and (**B**) AUC of DMXAA and (**C**) between *C*_max_ and percent free drug.

Peak plasma concentrations measured at the end of infusion increased from 4.8±3.1 *μ*M at 6 mg m^−2^ to 1290±105 *μ*M at 3700 mg m^−2^. The increase was linear with a slope of unity on a log–log plot up to 2300 mg m^−2^, but there was some evidence that concentrations reached a plateau at the highest two doses of 3700 and 4900 mg m^−2^, as shown in Figure 2A

.

The AUC increased nonlinearly with dose (slope 1.30±0.03) from 3.22±1.49 *μ*M h at the lowest dose (6 mg m^−2^) to 7640± 1610 *μ*M h at 3700 mg m^−2^ (Figure 2B).

At the highest dose level (4900 mg m^−2^), the AUC was higher than predicted from preclinical studies in the mouse. This may be in part due to interspecies differences in protein binding. At doses of up to 320 mg m^−2^, DMXAA was more than 99% plasma protein bound. At higher doses, the percentage of free drug increased, with a mean value of 4.2±1.4% above 1000 *μ*M. At the two highest doses (3700 and 4900 mg m^−2^),the mean free AUC was 2.31±0.16% of the total AUC. The relation between *C*_max_ and percent free drug is shown in Figure 2C. The nonlinearity of the AUC dose plot resulted from a gradual decrease in the clearance of DMXAA from 7.4±4.2 l h^−1^ m^−2^ at 6 mg m^−2^ to 1.65±0.39 l h^−1^ m^−2^ at 3700 mg m^−2^.

### Metabolites of DMXAA

The routes of DMXAA metabolism were initially identified from *in vitro* work in human liver microsomes (Webster *et al*, 1995; Miners *et al*, 1997). Several metabolites of DMXAA were identified by HPLC-MS in plasma from patients in this study, including 6-hydroxymethyl-5-methylxanthenone-4-acetic acid and glucuronides of 6-hydroxymethyl-5-methylxanthenone-4-acetic acid and the parent drug. Saturation of these metabolising pathways could account, at least in part, for the fall in clearance of DMXAA and the progressive change in the pharmacokinetics with increasing dose.

### Pharmacodynamic studies

**Figure 3:**
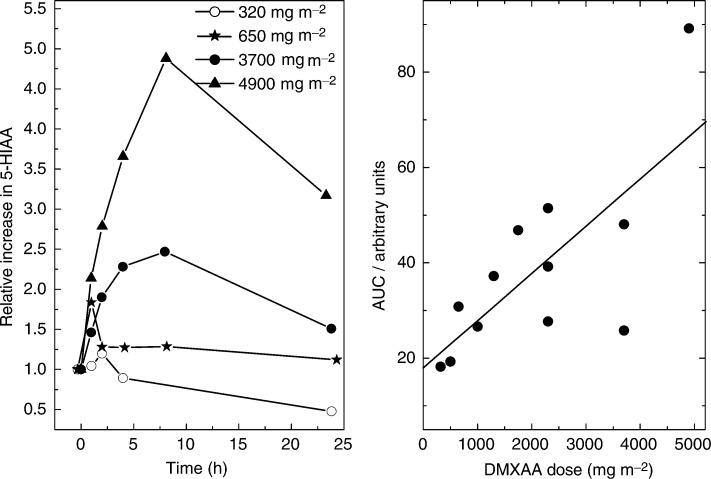
Effect of DMXAA dose on 5-HIAA plasma concentration and AUC.

There was a dose-dependent increase in plasma 5-HIAA at DMXAA doses of 650 mg m^−2^ and above, which became more marked both in extent and duration, as the dose of DMXAA increased, as shown in Figure 3

.

There was no significant increase in plasma TNF-*α* above control (preinfusion) levels and no consistent dose-related increases in plasma nitrate.

### Efficacy

Of the 46 patients treated with DMXAA, 36 were evaluated for their therapeutic response to treatment. In addition to the patient excluded because of a protocol violation (continued tamoxifen therapy), nine patients were either not assessed or an assessment of all lesions was not possible.

There was one unconfirmed partial response in a patient with locally recurrent melanoma treated at 1300 mg m^−2^ DMXAA. Prior to the study, this patient had undergone surgery, chemotherapy and hormonal but no immunotherapy or biotherapy. At 2 weeks after completing six infusions at this dose level, there was a reduction of more than 50% in measurable skin metastases. After 3 weeks, however, there was evidence of progressive disease and therefore this did not qualify as a partial response.

No change was recorded as the best response in six patients (13%) on a second tumour measurement performed after six infusions of DMXAA. However, none of these patients were considered for a further course because of lack of benefit.

## DISCUSSION

The early clinical trials of FAA were conducted before its indirect mechanisms of antitumour activity were identified and therefore took no account of cytokine induction. This phase I study not only examined the toxicity, MTD, and pharmacokinetic profile of DMXAA, but also measured the production of TNF-*α* and 5-HIAA, critical events in its antitumour activity (Philpott *et al*, 1995; Baguley *et al*, 1997). During the course of the study, the protocol was amended to include measurement of tumour blood flow before and after infusion of DMXAA, since reduction in tumour blood flow is central to its antitumour activity (Zwi *et al*, 1994a).

The tolerability of DMXAA was assessed at doses ranging from 6 to 4900 mg m^−2^. The results showed that DMXAA has a toxicity profile unlike that of conventional chemotherapy, with a notable absence of neutropenia, and no major cardiovascular effects. 5,6-Dimethylxanthenone acetic acid was generally well tolerated up to the highest dose levels, with patients experiencing the acute onset of symptoms including nausea and visual disturbance, the intensity and duration of which was largely dependent on dose. As visual disturbances had previously been reported in trials of FAA (Weiss *et al*, 1988; Kaye *et al*, 1990; Siegenthaler *et al*, 1992), once visual disturbances had been noted in this trial, patients were warned about this potential toxicity prior to therapy and found visual symptoms to be easily tolerable. Most importantly, each patient experienced a typical pattern of symptoms, which were the same after each course of treatment with DMXAA and rapidly reversible. The MTD was established at 3700 mg m^−2^.

This study showed that DMXAA has nonlinear (dose-dependent) pharmacokinetics and is highly protein bound in humans. Saturation of protein binding in combination with nonlinear pharmacokinetics resulted in a rapid increase in free plasma concentrations of DMXAA at dose levels above 650 mg m^−2^. As the dose of DMXAA increased almost 10-fold from 500 to 4900 mg m^−2^, the free plasma concentration increased more than 30-fold. The rapid increase in plasma concentrations at higher dose levels appears to correlate with increased toxicity.

There was a dose-dependent increase in the plasma concentration of 5-HIAA, the major metabolite of serotonin, at DMXAA dose levels of 650 mg m^−2^ and above, a change associated with vascular shutdown preclinically (Baguley *et al*, 1997; Kestell *et al*, 2001). Serotonin, probably released from platelets, has been suggested as a possible factor required for the vascular effect of DMXAA as the serotonin inhibitor cyproheptadine reduces the degree of induced haemorrhagic necrosis. There were no consistent dose-dependent increases in plasma nitrate or TNF-*α* the other factors implicated in the actions of DMXAA. This does not preclude an increase in tumour TNF-*α* levels, which might only be detected by examining tumour biopsies. Assessment of tumour blood flow by DCE-MRI, which was introduced to the study as a protocol amendment, did show a reduction in tumour blood flow at the lowest dose of DMXAA studied by this technique (500 mg m^−2^) (Galbraith *et al*, 2002). Only 11 patients had both serial 5-HIAA and DCE-MRI measurements and possibly because of the small numbers no relation between 5-HIAA changes and reduction in DCE-MRI kinetic parameters could be demonstrated. The clinically measured antitumour response seen in one patient in this study occurred at a dose of 1300 mg m^−2^ DMXAA. While it is clear that significant pharmacodynamic effects are occurring at intermediate and well-tolerated dose levels of DMXAA, further study is needed to assess whether there is a clinical advantage to using higher doses that would outweigh their greater toxicity.

Vascular targeting agents are able to induce extensive vascular shutdown and haemorrhagic necrosis in animal tumour models, but viable cells are often preserved in the peripheral rim and these cells will rapidly repopulate the tumour (Hill *et al*, 1991, 1992, 1993; Chaplin *et al*, 1999). This suggests that vascular targeting agents are unlikely to produce a clinical response when used as sole antitumour therapy. In this study, as in the parallel phase I study of DMXAA (Jameson *et al*, submitted), only a single unconfirmed tumour response was seen. Synergistic activity of DMXAA in combination with bioreductive cytotoxic drugs (Cliffe *et al*, 1994; Lash *et al*, 1998), chemotherapy (notably taxanes) (Pruijn *et al*, 1997; Horsman *et al*, 1999; Wilson and Baguley, 2000), thalidomide (Cao *et al*, 1999), immunotherapy (Kanwar *et al*, 2001), radiotherapy (Wilson *et al*, 1998), and radioimmunotherapy (Pedley *et al*, 1999) has been demonstrated in animal models, and the potential of DMXAA in cancer treatment with its novel mechanism of action is likely to lie in combination therapy with other treatment modalities.

In conclusion, this dose-escalation study demonstrates that DMXAA is well tolerated at doses with some effect on tumour blood flow. Although the MTD was 3700 mg m^−2^, it is planned to study more patients at lower doses to determine the lowest dose that reliably causes blood flow reduction before starting phase II trials. The lack of cumulative toxicity indicates that repeat dosing should be feasible. 5,6-Dimethylxanthenone acetic acid is a novel vascular targeting agent that should be studied further in clinical trials, particularly as a component of combination therapy.
